# Chronic immune activation and accelerated immune aging among HIV-infected adults receiving suppressive antiretroviral therapy for at least 12 years in an African cohort

**DOI:** 10.1016/j.heliyon.2024.e31910

**Published:** 2024-05-24

**Authors:** Damalie Nakanjako, Rose Nabatanzi, Isaac Ssinabulya, Lois Bayigga, Agnes Kiragga, Grace Banturaki, Barbara Castelnuovo

**Affiliations:** a Department of Medicine, School of Medicine, Makerere University College of Health Sciences, Kampala, Uganda; b Department of Immunology and Molecular Biology, School of Biomedical Sciences, Makerere University College of Health Sciences, Kampala, Uganda; c Uganda Heart Institute, Kampala, Uganda; d Infectious Diseases Institute, Makerere University College of Health Sciences, Kampala, Uganda

**Keywords:** Chronic inflammation, Immune activation, Immune-aging, HIV/AIDS, Non-communicable disease risk

## Abstract

**Background:**

HIV-associated alterations innate and adaptive immune cell compartments are reminiscent of the process of immune aging.

**Objectives:**

We described immune aging phenotypes among ART-treated HIV-infected adults relative to age-matched HIV-negative counterparts.

**Methods:**

In a cross-sectional comparative study of HIV-infected adults with CD4≥500 cells/μl after at least 12 years of suppressive ART and age-and-gender-matched HIV-negative individuals, immune activation and immune aging phenotypes were measured, using multi-color flowcytometry.

**Results:**

ART-treated HIV-infected individuals had higher body mass index (P = 0.004), waist-hip circumference (P = 0.041), hip circumference (P < 0.001), and diastolic blood pressure (P = 0.012) and immune activation (CD4^+^CD38+HLADR+; median 4.15,IQR(1.030,14.6)] relative to the HIV-negative age-matched individuals [median 3.14,IQR(1.030, 6.68)]; *P=0.0034.* Immune aging markers [CD4^+^CD57+T-cells; median 13.00 IQR (0.45,64.1)] were higher among HIV-infected ART-treated adults<50 years relative to HIV-negative<50 years[median 8.020,IQR(0.004,21.2)]; *P=0.0010*. Naïve CD4 T-cells, Central memory CD4 T-cells, Terminal Effector Memory T cells (TEMRA: CD27^−^CD45RA + CCR7-) and immune senescence CD4/CD8+CD28-/CD57+ T-cells were similar among ART-treated HIV-infected individuals<45 years relative to 60 years-and-older HIV-negative counterparts≥; p = 0.0932, p = 0.05357, p = 0.0950 and p = 0.5714 respectively**.**

**Conclusion:**

ART-treated adults are immunologically two decades older than their HIV-negative counterparts. Accelerated immune aging among individuals aging with HIV underscores the need for an HIV cure to avert the unprecedented complications of accelerated immune senescence and the associated NCD risk in African settings with protracted exposure to endemic co-infections.

## Highlights

• Immune aging markers were higher among HIV-infected ART-treated adults<50 years relative to HIV-negative adults <50 years.
• Naïve CD4 T-cells, Central memory CD4 T-cells, Terminal Effector Memory T cells and immune senescence were similar among ART-treated HIV-infected individuals<45 years relative to 60-years-and-older HIV-negative counterparts**.**
• ART-treated adults are immunologically two decades older than their HIV-negative counterparts.
• Accelerated immune aging among individuals aging with HIV underscores the need for an HIV cure to avert the unprecedented complications particularly in African settings with protracted exposure to endemic co-infections.
• We recommend well characterized studies to determine the specific contributions from each of the endemic infections, singly or in combination, towards the immune aging phenomenon among adults aging with HIV.

## 1 Introduction

Human aging exhibits significant changes in innate and adaptive immune responses [[1], [2], [3]]. There is increasing emphasis on aging with HIV due to the rapidly increasing numbers of HIV-infected individuals above 50 years [3,4]**.** HIV-associated alterations of the innate and adaptive immune cell compartments are reminiscent of the process of immune aging which is characteristic of old age [5,6]**.** Immune aging among HIV-infected adults has also been demonstrated by alteration of numbers and function of naïve T-cells due to significant reduction of thymus capacity to produce new T-cells [7,8], poor responses to inﬂuenza vaccination, and telomere shortening [9], that have been described among aging HIV-negative individuals [10,11]. Evidence suggests that chronic HIV-infection accelerates the immune changes that otherwise occur during normal aging [12,13]**.** Immune senescence and oxidative stress have been associated with frailty, a syndrome of physiological degeneration in the elderly living with HIV, in Europe and USA [14,15]**.** However, the extent to which HIV accelerates immune aging in Africans is not well understood in the context of persistent activation of the immune system due to protracted exposure to endemic pathogens including malaria, tuberculosis and helminths; among others.

The high levels of inflammation and immune activation associated with chronic HIV disease despite ART may contribute to accelerated immune aging and increase the risk of non-AIDS illnesses; including cardiovascular diseases [16,17], cataracts [18], malignancies [19,20], bone demineralization [21], renal disease [22] and cognitive decline [23] among HIV-positive adults relative to their HIV-negative counterparts. Activated T-cells (CD4/8+CD38+HLA-DR+) and CD28-/CD57+ T-cells have previously been used to demonstrate immune activation and senescence respectively [5,6], that were signiﬁcantly associated with increased risk of non-AIDS related multi-morbidities such as kidney disease, diabetes, dyslipidemia, cardiovascular events, hypertension, degenerative central nervous system disorders, and cancer among antiretroviral therapy **(**ART)-treated patients younger than 60 years [24]. CD57 expression on CD4^+^ and CD8 T-cells is a general marker of proliferative inability, a history of more cell divisions and short telomeres; resulting from chronic antigenic stimulation that is associated with chronic HIV infection; and thereby defines replicative senescence [25]. An increased proportion of CD8 T lymphocytes that lack CD28 expression has been associated additional features of replicative senescence [26]. Furthermore, increased frequencies of CD28-/CD57^+^ CD8+T-cells have been associated with the presence of Kaposi's sarcoma among successfully treated patients [27]. While CD57 is a good marker of senescence [5,6,26], the phenotype defined by CD28^−^CD57^+^ better characterizes the senescent cell. CD28 expression modulates telomere length and telomerase activity/activation, and is

Associated with shortening of telomeres and decreased telomerase activity [28]. However, CD28^−^ T cells retain some functionality, including the ability to up-regulate telomerase-dependent *p*-AktSer394 [29,30] and eventual loss of CD27 is a better correlate of reduced telomerase activity. Hence, loss of the coreceptors CD27 and/or CD28 indicates impaired telomere function in T-cells and progression towards replicative senescence. Similarly, it has been shown that the proportion of Terminal Effector Memory T cells (TEMRA: CD27^−^CD45RA + CCR7-) is a hallmark of immunosenescence [31,32]. Therefore, in this paper, we described immune aging as either of CD4/CD8 CD28^−^CD57^+^, CD4/CD8CD28-/CD57^+^ CD27^−^or CD27^−^CD45RA + CCR7- T-cells among adults receiving suppressive HIV treatment.

Even when ART was started within 18 days of HIV infection in the developed world, immune activation, as determined by sCD14, remained high after 96 weeks of therapy [33,34]**.** Within HIV treatment cohorts in sub-Saharan Africa (SSA), we demonstrated a high burden of persistent HIV-associated immune activation and inflammation during long-term suppressive ART among individuals that initiated ART after chronic HIV infection [35,36]**.** The synergistic effects of chronic HIV and co-infections, ART, and aging precipitate immune decline and the associated complex pathologies [7]**.** We previously demonstrated in our Ugandan community cohort that diseases of aging, such as blinding cataracts, occurred two decades earlier among HIV-infected ART-treated adults relative their HIV negative counterparts [18]. We therefore hypothesize that the chronic antigenic load among HIV-infected adults in SSA accelerates apoptosis and immune senescence [37]**;** with the most important characteristics as accumulation of memory and effector T-cells, reduction of naïve T-cells and shrinkage of T-cell repertoire [38]**.** This paper describes T-cell immune aging phenotypes among ART-treated individuals relative to age-matched HIV controls; and also compares immune aging phenotypes among HIV-infected adults below 45 years of age and HIV-negative adults that are approximately two decades older. Our results inform the global efforts to improve lives of people aging with HIV and ART through strategic interventions to delay immune aging processes and modify the associated risk of diseases of aging and non-comminicable diseases among adults aging with HIV in SSA. In addition, our results underscore the need to accelerate the search for an HIV cure to eradicate the latent HIV viral reservoirs that continue to drive the chronic immune activation, inflammation and aging among individuals receiving long-term suppressive ART.

## 2 Methods

Study setting and design: **This was a cross-sectional study, nested within the** IDI HIV treatment cohort of 345 active adults that have received ART for at least 12 years, within the HIV treatment cohort previously described by Kamya et al., 2007 [39]. Study participants were selected randomly from the 223 patients (out of 345) with a current CD4 count≥500 cells/μl, without opportunistic infections (OI) in the preceding 6 months, and with undetectable viral load (VL < 50 copies/ml).

Patient recruitment: HIV-infected ART treated adults, 18 years and over, with CD4 count≥500 cells/μl and VL < 50 copies/ml, and no co-infections (including Cytomegovirus, Hepatitis B virus, Hepatitis C virus, active tuberculosis and cryptococcosis) in the preceding six months were recuited consecutively, according to a computer-generated list of study numbers until the required sample size was attained. Participants were consented and recruited in the study according to their clinic appointments. Eligible participants without a scheduled clinic appointment during the study period were invited to the clinic by a phone call invitation. HIV-negative individuals were selected randomly from household members of the ART-treated individuals, and recruited through phone call contacts (given by the index client) or face-face invitation to the clinic by the study nurse-counselor (upon recommendation from the index patient). Written informed consent to participate in the study and storage of biological samples for future use was obtained by the study nurse-counselor. Study related clinical and laboratory procedures were similar for the ART-treated individuals and the comparative group of healthy HIV-negative individuals.

Clinical assessment: All patients were evaluated for clinical risk of CVD including biometrics height, weight, BMI, waist-hip ratio, presence and character of radial pulse and other pulses, and blood pressure. Full blood count, renal function tests, a pregnancy test for female clients and urinalysis were done as part of routine tests for HIV-infected and HIV-negative individuals.

### 2.1 Laboratory procedures

**Sample collection:** Eighty (80) mls of blood were collected for testing of comple blood count, C-reactive protein, d-dimers, renal function tests, fasting lipid profile, and peripheral blood mononuclear cells (PBMC) were processed using the Ficcol histopaque method according to already standardized protocols [36,40,41] in the translational immunology laboratories at Infectious Diseases Institute, Makerere University College of Health Sciences.

**Fig. 1:**
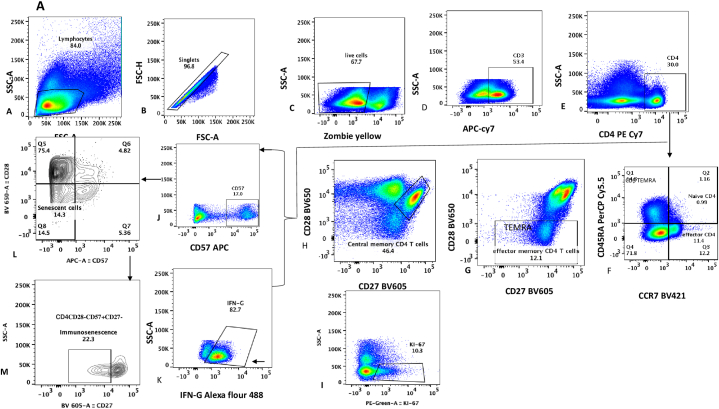

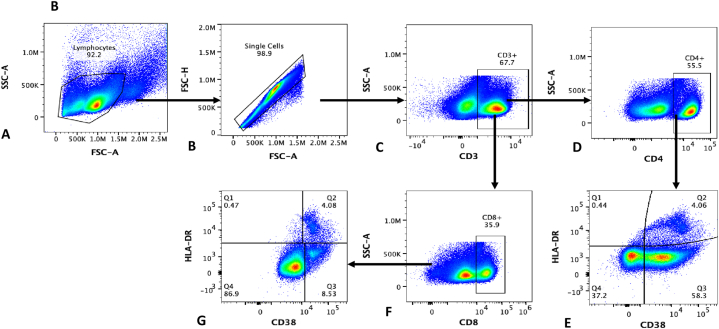
Gating strategy for Immune aging and immune activation among ART-treated individuaks: Figure1A: Gating strategy for Immune aging panel: A-shows lymphocytes, **B**-Singlets, **C**-Live gate, **D**- CD3 + T cells, **E**- CD4 + T cells, **F**- gating for Naïve and effector CD4 T cells **G**-effector memory CD4 T cells, **H**- shows Central memory CD4 T cells, **I**- Ki67 expression, J-percentage of CD4 expressing CD57, K-percentage of CD4 T cells producing interferon gamma, **L**-co-expression of CD57 and CD28 and **M**-percentage of CD4/CD8 T-cells CD28-/CD57^+^ CD27^−^Fig. 1B: Gating strategy that was used to determine immune activation profiles: A-shows the lymphocyte population, **B**-singlet gate, **C**-CD3 T-cells, **D**-CD4/CD8 T-cell gate off the CD3 T-cells, E,F,G-Immune activation as denoted by CD38 and HLADR positive cells.

**PBMC Separation and immunophenotyping:** Frozen PBMCs were thawed and batch analysed for immune activation and immune aging markers using flow cytometry acquired on Cytex Aurora flowcytometer. Numbers and phenotypes of T-cells were analysed using the gating strategy in Fig. 1 (A-B). Immune activation was measured by the proportion of CD4/CD8 T-cells co-expressing cell surface markers CD38 and HLADR (CD4+/CD8+CD38+HLADR+) and immune aging was determined by high proportion of CD4/CD8 T-cells expressing CD57 (CD3^+^CD4+/CD8+CD57+T-cells), proportion of CD4/CD8CD28-/CD57^+^ CD27^−^ T-cells and proportion of Terminal Effector Memory T-cells (TEMRA: CD3^+^CD4/CD8+CD45RA + CCR7– T-cells). T-cell proliferation was described by high proportion of T-cells producing Inferon gama (CD3^+^CD4+/CD8+IFN-ɣ+) and CD4^+^ (CD8^+^) Ki 67 expression. Additional correlates of aging described included high proportion of Central memory T-cells (CD3^+^CD4/CD8+CD45RA–CCR7+CD27^+^CD28^+^) as well as low proportions of Naïve T-cells (CD3^+^CD4/CD8+CD45RA + CCR7+CD8+T-cells).

**Data analysis:** We analysed the proportion of CD4/CD8 T-cells expressing immune aging, immune activation and T-cell proliferation markers using the gating strategy illustrated in Fig. 1. Flowcytometry data was analysed using flowjo and PRISM graphpad for comparisons between ART-treated individuals below 50 years and their age-matched HIV-negative counterparts. Students’ T-test and the Kruskal-Wallis test were used for non-parametric variables. Parameters were compared among ART-treated adults and healthy age-matched HIV-negative individuals, with statistical significance at *P*-value<0.05. In addition, comparisons were made between HIV-infected individuals aged 45 years and below and HIV negative individuals 60 years and over to contribute to the understanding of why diseases of aging such as blinding cataracts in sub-Saharan Africa occurred two decades earlier among HIV-infected adults relative to their HIV-negative counterparts. Logistic regression was done to determine clinical and laboratory predictors of non-communicable disease risk among individuals receiving suppressive ART for a least 12 years and their age-matched HIV-negative counterparts from the same community (as a reference group).

Ethical consideration: This study was approved by the research and ethics committee and the Joint Clinical research Centre and subsequently approved by the Uganda national council for science and technology (HS194ES). All participants provided written informed consent to participate in the study and to have their biological samples stored for future studies to understand immune recovery during HIV treatment in an African cohort.

## 3 Results

Socio-demographic characteristics of HIV-infected adults with at least 12 years of suppressive ART and HIV-negative comparative group from the same community of the IDI HIV treatment cohort, were similar (Table 1). Similarly family history of non-communicable disease risk was comparable, although physical examination showed that ART-treated HIV-infected individuals had higher body mass index (p = 0.004), waist-hip circumference (p = 0.041), hip circumference (p < 0.001), and diastolic blood pressure (p = 0.012); see Table 2.

**Table 1:** Socio-demographic characteristics of HIV-infected adults with at least 12 years of suppressive ART and HIV-negative comparative group from the same community of the IDI HIV treatment cohort, in Uganda.

	HIV negative	HIV positive	Total	p-value
	(N = 72)	(N = 188)	(N = 260)	
Age				0.059
Median (Q1, Q3)	47.0 (41.0, 55.0)	50.0 (45.0, 55.0)	49.0 (44.0, 55.0)	
**Sex**				0.439
Female	52 (73.2 %)	127 (68.3 %)	179 (69.6 %)	
**Marital Status**				
Never married	8(11.2 %)	17(9.1 %)	25(9.7 %)	0.006
Married/Cohabiting	42(59.2 %)	78(41.9 %)	120(46.7 %)	
Divorced	14(19.7 %)	34(18.3 %)	48(18.7 %)	
Widowed	7(9.9 %)	57(30.7 %)	64(24.9 %)	
**Occupation**				0.509
Unemployed	4 (5.6 %)	21 (11.4 %)	25 (9.7 %)	
Student	0 (0.0 %)	3 (1.6 %)	3 (1.2 %)	
Employed	51 (70.8 %)	119 (64.3 %)	170 (66.1 %)	
Retired	1 (1.4 %)	6 (3.2 %)	7 (2.7 %)	
Peasant	16 (22.2 %)	36 (19.5 %)	52 (20.2 %)	
**Education**				0.245
Never attended	10 (13.9 %)	33 (17.8 %)	43 (16.7 %)	
Primary	27 (37.5 %)	78 (42.2 %)	105 (40.9 %)	
Secondary	21 (29.2 %)	55 (29.7 %)	76 (29.6 %)	
Post-secondary	14 (19.4 %)	19 (10.3 %)	33 (12.8 %)	
**Drinking alcohol**				0.003
Yes	30 (41.7 %)	43 (23.0 %)	73 (28.2 %)	
**Smoke cigarettes**				0.194
Yes	6 (8.5 %)	8 (4.3 %)	14 (5.5 %)	
**Active during leisure time**				0.895
Maintain sedentary lifestyle	14 (19.7 %)	41 (22.5 %)	55 (21.7 %)	
Mild exercise (minimal effort)	26 (36.6 %)	60 (33.0 %)	86 (34.0 %)	
Moderate exercise	27 (38.0 %)	73 (40.1 %)	100 (39.5 %)	
Strenuous	4 (5.6 %)	8 (4.4 %)	12 (4.7 %)

**Table 2:** Non-communicable disease risk among HIV-infected adults with at least 12 years of suppressive ART and HIV-negative comparative group from the same community of the IDI HIV treatment cohort, in Uganda.

	HIV negative	HIV positive	Total	
	(N = 72)	(N = 188)	(N = 260)	p-value
Family history of non-communicable disease risk				
**Hypertension**				0.632
Yes	34 (47.2 %)	97 (52.2 %)	131 (50.8 %)	
No	34 (47.2 %)	82 (44.1 %)	116 (45.0 %)	
Don't know	4 (5.6 %)	7 (3.8 %)	11 (4.3 %)	
**Diabetes**				0.341
Yes	27 (37.5 %)	52 (28.0 %)	79 (30.6 %)	
No	41 (56.9 %)	122 (65.6 %)	163 (63.2 %)	
Don't know	4 (5.6 %)	12 (6.5 %)	16 (6.2 %)	
**Obesity/Overweight**				0.994
Yes	24 (33.3 %)	63 (34.1 %)	87 (33.9 %)	
No	46 (63.9 %)	117 (63.2 %)	163 (63.4 %)	
Don't know	2 (2.8 %)	5 (2.7 %)	7 (2.7 %)	
**Sudden death**				0.475
Yes	9 (12.7 %)	16 (8.7 %)	25 (9.8 %)	
No	57 (80.3 %)	159 (86.4 %)	216 (84.7 %)	
Don't know	5 (7.0 %)	9 (4.9 %)	14 (5.5 %)	
**Stroke**				0.473
Yes	17 (23.9 %)	33 (17.8 %)	50 (19.5 %)	
No	50 (70.4 %)	137 (74.1 %)	187 (73.0 %)	
Don't know	4 (5.6 %)	15 (8.1 %)	19 (7.4 %)	
Physical examination assessment of non-communicable disease risk				
Body Mass Index (BMI)				
≤18.5	4(6.0 %)	27(15.0 %)	31(12.6 %)	0.004
18.6–24.9	27(40.3 %)	96(53.3 %)	123(49.8 %)	
≥25	36(53.7)	57(31.7 %)	93(37.7 %)	
**Neck circumference**				0.010
Median (Q1, Q3)	36.0 (34.0, 38.0)	35.0 (33.0, 37.0)	35.0 (33.0, 37.0)	
**Waist circumference**				0.041
Median (Q1, Q3)	90.5 (82.0, 97.0)	85.6 (79.7, 94.0)	86.9 (80.0, 95.0)	
**Hip circumference**				<0.001
Median (Q1, Q3)	109.0 (99.7, 115.3)	98.0 (93.0, 106.2)	100.8 (94.0, 109.0)	
**Waist to hip ratio**				0.309
Median (Q1, Q3)	0.8 (0.8, 0.9)	0.9 (0.8, 0.9)	0.9 (0.8, 0.9)	
**Systolic blood pressure**				0.085
Median (Q1, Q3)	122.0 (110.0, 134.0)	117.0 (108.0, 129.0)	118.0 (108.0, 131.0)	
**Diastolic blood pressure**				0.012
Median (Q1, Q3)	79.0 (71.5, 88.0)	77.0 (70.0, 82.0)	78.0 (71.0, 84.0)	

**Fig. 2:**
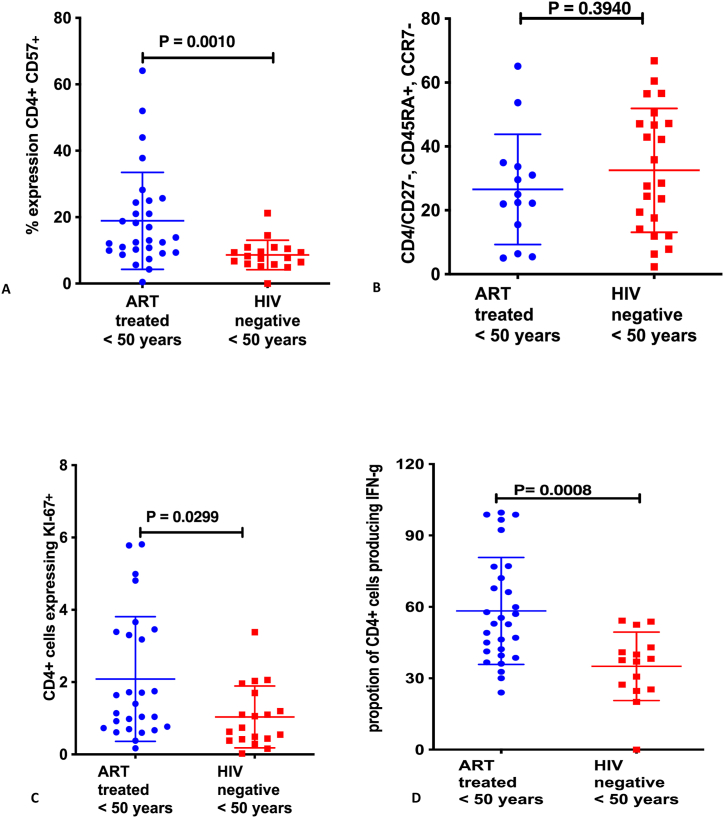
Immune aging phenotypes and T-cell proliferation among ART-treated HIV-infected individuals below 50 years and their age-matched HIV-negative counterparts: A shows proportion of CD4^+^ T-cells expressing CD57, **B** shows Terminal Effector Memory T cells (TEMRA: CD27^−^CD45RA + CCR7-), **C** shows proportion of CD4^+^ T-cells expressing Ki67, and **D** shows proportion of CD4 T-cells producing interferon gamma.

**Immune activation, immune aging and T-cell proliferation among ART-treated HIV-infected individuals below 50 years and their age-matched HIV-negative counterparts:** Overall, immune activation was higher among ART-treated HIV-infected adults [CD4^+^CD38+HLADR+; median 4.15, IQR (1.030, 14.6)] relative to the HIV-negative age-matched individuals from the same community [CD4^+^CD38+HLADR+; median 3.14, IQR (1.030, 6.68)]; (p = 0.0034). The proportion of CD4 T-cells expressing immune aging marker CD57 was higher among HIV-infected ART-treated adults below 50 years [CD4^+^CD57+T-cells; median 13.00, IQR (0.45, 64.1)] relative to their age-matched HIV-negative counterparts [median 8.020, IQR (0.004, 21.2)]; p = 0.0010. Similarly CD4 T-cell proliferation was higher among ART-treated HIV-infected adults <50 years relative to their age-matched HIV-negative comparative group; CD4+Ki67+T-cells, median 1.400, IQR (0.1700, 5.81) among ART-treated HIV-infected versus median 0.740, IQR (0.0250, 3.38)] among age-matched HIV-negative adults; p = 0.0299 and CD4+T-cell production of interferon gamma production of median 52.9, IQR (24.00, 99.6)] among ART-treated HIV-infected individuals <50 years relative to age-matched HIV-negative individuals with median 37.6, IQR (0.037, 54.2)]; p = 0.0008 [Fig. 2 (A-D)].

**Fig. 3:**
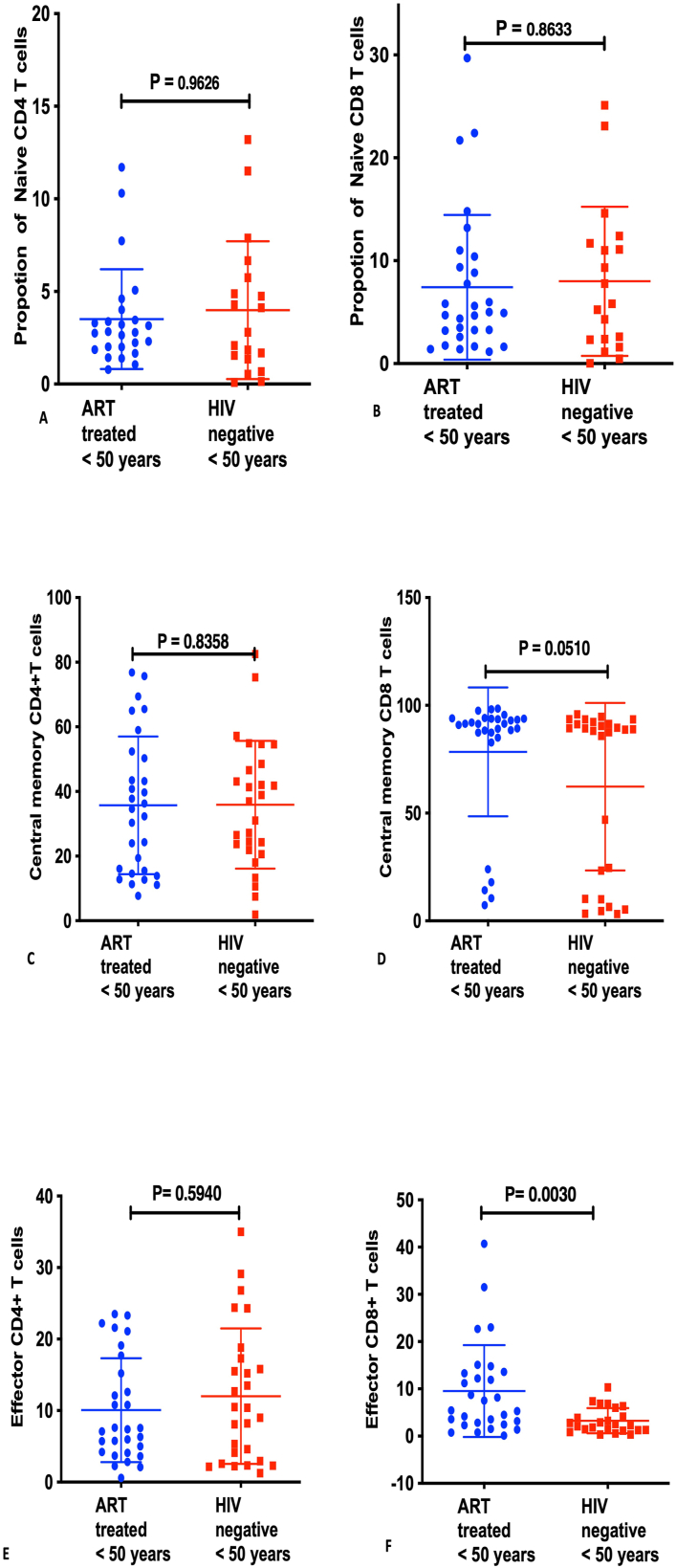
T-cell phenotypes among ART-treated HIV-infected individuals below 50 years and their age-matched HIV-negative counterparts: A shows Naive CD4+T-cells**, B** shows CD8^+^ naïve T-cells**, C** shows Central memory CD4^+^ T-cells, **D** shows Central memory CD8^+^ T-cells, **E** shows Effector CD4^+^ T-cells and **F** shows Effector CD8^+^ T-cells.

**CD4/CD8 T-cell subsets among ART-treated individuals <50 years and their age-matched HIV-negative counterparts:** In terms of numbers/frequency of T-cell phenotypes, proportions of effector CD8+Tcells were higher among HIV-infected ART-treaed adults (P = 0.0030), although the other phenotypes were comparable in the two groups [Naïve CD4 T-cells groups [Naïve CD4 T-cells [median 2.79, IQR (0.78, 11.7)] among HIV-infected ART-treated versus HIV-negative [median 2.80, IQR (0.085, 13.2)]; p = 0.9626, Naïve CD8 T-cells among HIV-infected ART-treated [median 4.92, IQR (1.16, 29.7)] versus HIV-negative [median 5.82, IQR (0.031, 25.1)]; p = 0.8633; and Central memory CD4 T-cells among HIV-infected ART-treated [median 34.6, IQR (7.69, 76.8)] versus HIV-negative [median 36.9, IQR (1.90, 82.5)]; p = 0.8358 and Central memory CD8 T-cells among HIV-infected ART-treated [median 91.4, IQR (7.27, 98.5)] versus HIV-negative [median 88.7, IQR (3.20, 95.8)]; p = 0.0510 **[**Fig. 3 (A-F)].

**Fig. 4:**
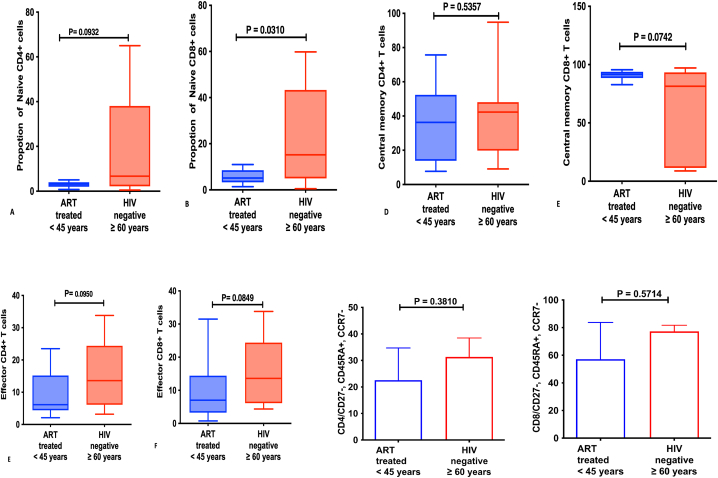
T-cell phenotypes among ART-treated HIV-infected individuals below 45 years and their 60 years-and-older HIV-negative counterparts: **A** shows Naive CD4+T-cells**, B** shows CD8^+^ naïve T-cells**, C** shows Central memory CD4^+^ T-cells, **D** shows Central memory CD8^+^ T-cells, **E** shows Effector CD4^+^ T-cells, **F** shows Effector CD8^+^ T-cells, **G** shows Terminal Effector Memory T cells (CD4^+^CD27^−^CD45RA + CCR7-) and **H** shows CD4^+^CD27^−^CD45RA + CCR7-.

**CD4/CD8 T-cell subsets among ART-treated HIV-infected individuals below 45 years and 60 years-and-older HIV-negative counterparts.** Proportions of Naïve CD4 T-cells, Central memory CD4 T-cells, Effector CD4 T-cells and Terminal Effector memory CD4 T-cells were similar among ART-treated HIV-infected individuals below 45 years relative their 60 years-and-older HIV-negative counterparts; p = 0.0932, p = 0.0535, p = 0.0950 and 0.5714 respectively [Fig. 4 (A-H)].

Furthermore, expression of immune aging markers and T-cell proliferation among ART-treated HIV-infected individuals below 45 years was comparable to their 60-years-and-older HIV-negative counterparts. The proportion of CD4 T-cells expressing CD57 and CD4/CD8+CD28^−^CD57^+^ T-cells

**Fig. 5:**
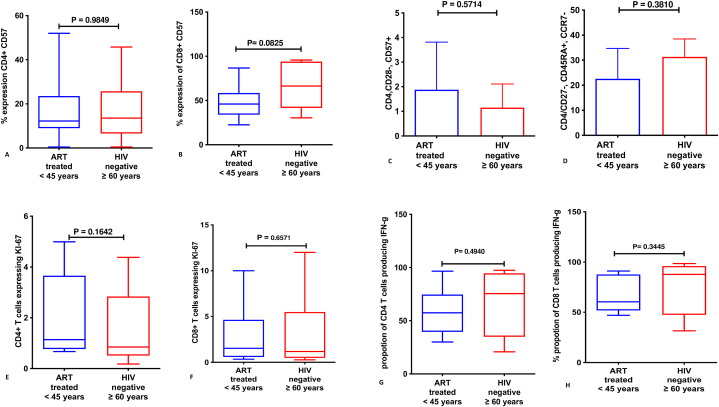
Immune aging markers and T-cell proliferation among ART-treated HIV-infected individuals below 45 years and their 60-years-and-older HIV-negative counterparts: A shows proportion of CD4^+^ T-cells expressing CD57, **B** shows proportion of CD8 +T-cells expressing CD57, **C** shows proportion of CD4^+^CD28^−^CD57^+^ T-cells, **D** shows CD4^+^CD27^−^CD45RA + CCR7- (Terminal Effector Memory T-cells), **E** shows proportion of CD4^+^ T-cells expressing Ki67, and **F** shows proportion of CD8^+^ T-cells expressing Ki67, **G** shows proportion of CD4 T-cells producing interferon gamma and **H** shows proportion of CD8 T-cells producing interferon gamma.

Were comparable among HIV-infected individuals below 45 years and their 60-years-and-older HIV-negative counterparts; CD4^+^CD57^+^ T-cells; median 12.25,IQR(0.4,52.00) versus CD4^+^CD57^+^ T-cells; median 13.55, IQR (0.460, 45.80)]; p = 0.9849 and CD8^+^CD57^+^ T-cells median 46.00, IQR (22.5, 86.7)] among ART-treated HIV-infected individuals below 45 years versus median 66.35, IQR (30.4, 95.7)] among their 60-years-and-older HIV-negative counterparts; p = 0.0825. In addition, T-cell proliferation (CD4+/CD8+Ki67+) was comparable among ART-treated HIV-infected individuals below 45 years [median 1.14, IQR (0.67, 4.99)] and their 60-years-and-older HIV-negative counterparts [median 085, IQR (0.18, 4.38)]; p = 0.1642) and CD8KI67+ median 1.53, IQR (0.34, 10.00) among infected individuals below 45 years versus median 1.18, IQR (0.27, 12.00)] among their 60-years-and-older HIV-negative counterparts; p = 0.6571 [Fig. 5 (A-H)].

## 4 Discussion

Our data illustrate that ART-treated HIV-infected adults below 50 years had higher expression of immune aging markers relative to their age-matched HIV-negative counterparts; despite suppressive ART and restoration of CD4 counts to levels ≥500 cells/μl. Similarly, the expression of immune aging markers among ART-treated HIV-infected adults below 45 years was similar to that of their HIV-negative counterparts who were two decades older. Both of these results provide evidence of accelerated immune aging among HIV-infected individuals despite suppressive ART. Accelerated immune aging among HIV-infected adults predisposes them to early occurrence of diseases of aging including non-communicable diseases, which are increasing as causes of morbidity and mortality among adults living with HIV in the ART-era. Our findings are consistent with our previous reports that blinding cataracts occurred two decades earlier among HIV-infected adults relative to their HIV-negative counterparts with blinding cataracts in an Ugandan community cohort [18]**;** a phenomenon that was associated with high levels of immune activation and inflammation and low levels of regulatory T-cells among the HIV-infected individuals [5,18,42]. Given the high immune activation and regulatory T-cell dysfunction we described among HIV-infected adults with blinding cataracts relative to their HIV-negative counterparts with the same, we proposed further research to understand the role of immune modulation interventions as strategies to delay development of cataracts [18]**.** There is yet no consensus on whether HIV-infected adults develop HIV-associated cataracts because chronic HIV disease accelerates aging or because HIV infection and ART are additive risk factors just like age, smoking and use of glucocorticoids.

Our finding of lower proportion of naïve T-cells among the aging HIV-infected population is consistent with previous reports of increased turnover and reduced frequency of naïve T-cells among the elderly [43,44]. Reduced numbers and function of naïve T-cells is likely due to significant reduction of thymus capacity to produce new T-cells, particularly in the setting of HIV infection when both naive and memory T cells are progressively depleted [45]. Our results are consistent with the decline in naive T-cell populations that has been reported in HIV treatment cohorts in Europe as a marker of premature development of an immunosenescence phenotype with HIV disease progression [46]**.**

HIV-associated inflammation and immune dysfunction have given way to chronic diseases, that are emerging as leading causes of death of people aging with HIV in the long-term ART era; hence the need to further understand not only the onset of immunosenescence among ART-treated individuals, but also the deterioration of several physiological functions related to inflammation and systemic aging [47]**.** Emerging data suggests that immunologic aging processes among HIV-infected individuals include accumulation of terminal stage CD8 T-cells and limited T-cell proliferation potential, which have been associated with clinical problems and frailty among individuals aging with HIV [2,18,48]. Moreover, immune dysfunction and inflammation concomitant with viral infections and multi-morbidity have been associated with premature functional decline, susceptibility to additional illnesses and mortality [18,49]. Therefore, the comparison between HIV-infected patients and uninfected elderly adults goes beyond the sole onset of immunosenescence and extends to the deterioration of a number of physiological functions related to inflammation and systemic aging [6,43]. A better understanding of the precise link between immune activation, aging and non-communicable disease risk during chronic HIV infection is necessary to inform interventions to manage or delay aging processes and related complications among people living with HIV [50].

HIV-associated premature immune aging may be prevented or delayed among young HIV-infected infants and adolescents, particularly in SSA which has a high burden of peri-natal HIV transmission and adolescents living with HIV [51]**.** Studies with long-term HIV treatment cohorts in Europe showed that understanding the immune mechanisms underlying HIV-associated inflammaging, immune aging and frailty status in HIV-1 populations is of high relevance not only for the prediction of continuing longevity but also for the identification of potential strategies for management of the elderly [15]**.** This is even more apparent in SSA where the host immune system already faces immunological insults from protracted exposure to endemic infections including malaria, tuberculosis, and intestinal helminths; among others, and where the concept of aging is least understood due to previous centuries of low life-expectancy in these low-and middle income countries [52].

The naïve CD8 T-cell population was smaller among HIV-infected ART-treated adults below 45 years relative to HIV-negative individuals 60 years and older, reflecting faster aging process among the HIV-infected individuals. In aged mice, the dwindling naïve CD8^+^ T-cells are key players in immune response against pathogens and tumors. Reduced CD8+T-cell priming capacity in-vitro was further associated with poor immune responsiveness in-vivo, as a likely consequence of intrinsic cellular defects and a reduction in the size of naïve T-cell pool which accompanies human aging [53]**.** Previous studies in humanized mice have demonstrated that HIV disease progression correlates positively with generation of dysfunctional naïve CD8 low T-cells [54] as a result of IFN α mediated upregulation of MHC-1 on stromal cells in the thymus and antigen-presenting cells in the periphery. Moreover, the dysfunction in the naïve T-cell compartment contributes to the immunodeficiency of HIV disease [54]. We, however, did not differentiate lineage of the CD4 T-cells to validate the findings from in-vitro studies which showed that CD4 T-cells from the A18 TCR transgenic strain normally selected into the CD4 lineage are fragile as CD4 T-cells although they display a typical robust survival pattern of CD8 T-cells when diverted into the CD8 lineage in a CD4 deficient host [55].

Furthermore, we demonstrated high IFNβ production among ART-treated adults relative to their age-matched HIV-negative counterparts. Similarly, the IFNβ production of T-cells from ART-treated adults below 45 years was comparable to that of HIV-negative adults who were two decades older. In the absence of pathogen infection, host-derived nucleic acids may activate the interferon pathway abberrantly, a process that is now postulated to take place during aging [56]. DNA damage in the bonemarrow stem cells can induce IFNβ production which leads to immune stem cell senescence and decline [1]**.**

### 4.1 HIV associated non-communicable diseases (NCD) risk

Chronic HIV infection has been associated with increased risk of NCD including acute coronary syndrome which occurred a decade earlier among HIV-infected patients relative to their HIV-negative counterparts that were hospitalized with acute coronary syndromes at San Francisco General Hospital from 1993 to 2003 [57] and increased carotid intima-media thickness among individuals with increased cytomegalovirus-specific T-cell responses in an ongoing clinic-based cohort based at the University of California, USA [58]**.** HIV-associated NCD risk is likely higher in SSA due to the protracted exposure to other infectious agents (including the endemic malaria, tuberculosis and helminths); for example, we previously reported high inflammatory markers and occurrence of blinding cataracts (requiring surgery) two decades earlier among HIV-infected ART-treated adults relative to HIV-negative counterparts with blinding cataracts in an Ugandan community cohort [18,42]. Although ART-treated HIV-infected adults in our cohort had similar NCD risk to their HIV-negative counterparts (as evaluated from the sociodemographic and medical history), physical examination revealed higher risk of NCD risk (as determined by higher body mass index, waist-hip ratio, hip circumference and diastolic blood pressure) which may be associated with the long-term side effects of ART and/or HIV-associated persistent immune activation, inflammation and immune senescence.

### 4.2 Modulation of NCD risk among ART-treated HIV-infected individuals

With over 20.1 million people living with HIV receiving ART in SSA, more HIV-infected patients are surviving with over 20 years of ART and may require modification of HIV associated aging as well as NCD morbidity and mortality. These data highlight the need to consider adjunct therapy to reduce immune activation among ART-treated adults; for example statins [59,60], probiotics [61] and senolytics [62]**.** Furthermore, agents that inhibit or reverse immunesenesce may be considered in African cohorts; such as inhibition of p38 MAPK in senescent CD8^+^ T-cells which was shown to increase T-cell proliferation, telemerase activity, mitochondrial biogenesis and fitness [63]**,** Vitamin D supplementation which has been shown to reduce immune activation levels among ART-treated HIV-infected individuals [64]**,** Sitaglipptin which was shown to reduce plasma high-sensitivity C-reactive protein as well as C-X-C motif chemokine and Rifaximin, a luminal antibiotic that attenuated T-cell activation [65] among HIV-infected individuals with impared glucose tolerance [66]**.** Ultimately, our data underscore the need to fast track the search for an HIV cure in SSA because chronic HIV infection and long-term suppressive ART clearly remain with unprecedented complications of accelerated immune-senescence and the associated NCD risk, within the context of host immune activation due to endemic infections in Africa. This study did not measure p16 (INK4a) expression which reflects both cellular senescence and biologic aging in almost all organs [67] and telomere shortening since short telomeres trigger DNA damage checkpoints that cause mitotic arrest and cell senescence [9]. It is important to note that the immunosenescence markers measured in this study have been correlated with reduced activity of telomerase [68]. Another limitation of this study was the inability to correlate immune aging parameters with specific antiretroviral drugs since all study participants had received Nucleoside Reverse Transcriptase Inhibitor (NRTI), Non-Nucleoside Reverse Transcriptase Inhibitor (NNRTI), Protease inhibitors (PI), and Integrase Strand Transfer Inhibitor (INSTI) since 2004 based on the national treatment guidelines [39]. We further recommend well characterised studies to understand specific contributions of each antiretroviral drug to the immune aging phenomenon in sub-Saharan African HIV treatment cohorts to inform strategic interventions to counteract HIV-and-ART-associated immune aging and its complications [62,69,70].

## 5 Conclusion

Immune aging markers were higher among ART-treated HIV-infected individuals below 45 years relative to their HIV-negative counterparts, and comparable to HIV-negative individuals two decades older. Accelerated immune aging underscores the need to fast track the search for an HIV cure in SSA to avert the unprecedented complications of accelerated immune senescence and the associated NCD risk in African settings where most individuals have background immune activation due to protracted exposure to endemic infections. We recommend well characterised studies to determine the specific contributions from each of the endemic infections, singly or in combination, towards the immune aging phenomenon among adults aging with HIV.

## Consent for publication

Not applicable.

## Availability of data and materials

All data will be made available upon request.

## CRediT authorship contribution statement

**Damalie Nakanjako:** Writing – review & editing, Writing – original draft, Visualization, Validation, Supervision, Software, Resources, Project administration, Methodology, Investigation, Funding acquisition, Formal analysis, Data curation, Conceptualization. **Rose Nabatanzi:** Writing – review & editing, Validation, Methodology, Investigation, Formal analysis, Data curation. **Isaac Ssinabulya:** Writing – review & editing, Visualization, Validation, Project administration, Methodology, Investigation, Data curation. **Lois Bayigga:** Writing – review & editing, Validation, Methodology, Investigation, Data curation, Conceptualization. **Agnes Kiragga:** Writing – review & editing, Validation, Methodology, Formal analysis, Data curation. **Grace Banturaki:** Writing – review & editing, Validation, Methodology, Investigation, Formal analysis. **Barbara Castelnuovo:** Writing – review & editing, Validation, Resources, Methodology, Investigation, Conceptualization.

## Declaration of competing interest

The authors declare that they do not have any competing interests.
